# Treatment and Survival for Unresectable Pancreatic Adenocarcinoma in Queensland, Australia, 2018–2022

**DOI:** 10.1002/cam4.71226

**Published:** 2025-09-08

**Authors:** Danny R. Youlden, Bryan A. Chan, Jon Clark, Victoria K. Donoghue, Michael J. Allen

**Affiliations:** ^1^ Cancer Alliance Queensland Metro South Health Brisbane Queensland Australia; ^2^ Adem Crosby Cancer Centre, Department of Medical Oncology, Division of Cancer Care Services Sunshine Coast University Hospital Birtinya Queensland Australia; ^3^ School of Medicine and Dentistry Griffith University Birtinya Queensland Australia; ^4^ Centre for Bioinnovation University of the Sunshine Coast Sippy Downs Queensland Australia

**Keywords:** chemotherapy, pancreatic cancer, Queensland, survival

## Abstract

**Background:**

The three main chemotherapy regimens for people with unresectable pancreatic cancer include modified FOLFIRINOX (comprising oxaliplatin, irinotecan and fluorouracil, denoted mFFX), gemcitabine with nab‐paclitaxel (GnP), and single‐agent gemcitabine (GEM). We explored characteristics associated with the type of chemotherapy and variations in survival.

**Materials and Methods:**

Records for people with unresected pancreatic adenocarcinoma between 2018 and 2022 treated with first‐line mFFX, GnP or GEM were extracted from the population‐based Queensland Oncology Repository. Multivariable Poisson models were fitted to determine factors associated with each type of chemotherapy, expressed as relative likelihoods (RLs). Variations in three‐year observed survival were assessed using flexible parametric modelling and reported in terms of adjusted excess mortality hazard ratios (HRs).

**Results:**

Of the 766 people in the study cohort, 59% were treated with GnP, 27% with mFFX, and 15% with GEM. After adjustment, treatment with mFFX was far more likely in selected private facilities compared to public hospitals (RL = 2.33, 95% CI 1.84–2.96), whereas the GEM regimen was used more often for those from outer regional/remote areas (RL = 2.20 compared to people living in major cities, 95% CI 1.45–3.34; *p* < 0.001). Three‐year survival was very poor at just 5% (95% CI 3%–7%). Nonetheless, adjusted mortality was higher for GnP (HR = 1.30, 95% CI 1.07–1.59) and GEM (HR = 1.53, 95% CI 1.17–2.01) compared to mFFX.

**Conclusions:**

Apart from clinical indications, there should be equity in the treatment received for unresectable pancreatic cancer. Our results suggest, however, that where a person lives and the type of facility at which they are treated may influence their chemotherapy regimen.

**Abbreviations**
CI: confidence interval
GEM: single‐agent gemcitabine
GnP: gemcitabine with nab‐paclitaxel
HR: hazard ratio
ICD‐O: International Classification of Diseases for Oncology
IQR: interquartile range
mFFX: modified FOLFIRINOX
QOR: Queensland Oncology Repository
RL: relative likelihood

## 1 Introduction

Pancreatic cancer has one of the highest incidence‐to‐mortality ratios of any malignancy. It is estimated that there were 511,000 new cases and 467,000 deaths due to pancreatic cancer worldwide in 2022, equating to 5% of all cancer‐related deaths [1]. The number of diagnoses climbs sharply as age increases, with more than two‐thirds of cases (68%) occurring in people over 65 years old [2]. The majority of cases are classified as adenocarcinoma subtype (~90%) [3] which has distinctive molecular and behavioural characteristics compared to other types of pancreatic cancer [4, 5, 6, 7], most notably rapid progression and early spread or metastasis. Although the disease burden remains greatest in high‐income regions such as North America, Western Europe and Australia, incidence rates of pancreatic cancer have been rising rapidly throughout the Caribbean, Latin America and Asia over recent decades [8].

The low survival rate for pancreatic cancer is primarily due to the asymptomatic nature of the disease in its early stages, leading to diagnoses predominantly occurring at a late stage when curative treatment options are limited [4, 9]. Complete resection with clear margins forms the cornerstone of curative treatment for pancreatic cancer, [10] but only 15%–20% of cases are diagnosed at an early stage where surgical resection is possible [11, 12]. Even with surgical intervention, recurrence rates are high and long‐term survival remains low [13, 14].

Chemotherapy is used as the primary treatment for people with unresectable disease, the intent being to extend life expectancy and/or improve symptom control with palliative intent. There are three main chemotherapy regimens funded by the Pharmaceutical Benefits Scheme and currently used in Queensland within the palliative setting: oxaliplatin, irinotecan and fluorouracil (modified FOLFIRINOX, denoted mFFX); gemcitabine with nab‐paclitaxel (GnP); and single‐agent gemcitabine alone (GEM) [15]. These regimens have varying levels of effectiveness and tolerance, with mFFX and GnP both reported to provide longer median survival than GEM, but with the potential offset of greater toxicity [16, 17].

The main aim of this study was to investigate whether any key variables influence the type of first‐line chemotherapy for unresectable/metastatic pancreatic cancer after accounting for clinical variables such as stage at diagnosis and comorbid conditions, including location of residence, socio‐economic status, and differences between private and public oncology care. We also evaluated survival for the three main treatment regimens.

## 2 Methods

### 2.1 Data

People included in the study cohort were residents of Queensland, Australia, who were diagnosed with unresectable adenocarcinoma of the pancreas (ICD‐O site codes C25.x and ICD‐O morphology codes 8140/3, 8480/3, 8490/3 and 8500/3) between 2018 and 2022 and received one of the three main chemotherapy regimens (mFFX, GnP or GEM), either with or without radiotherapy. Unit record data were obtained from the population‐based Queensland Oncology Repository (QOR) in accordance with Section 82 of the *Hospital and Health Boards Act* (2011) (see https://www.legislation.qld.gov.au/view/whole/html/inforce/current/act‐2011‐032), thereby exempting the study from ethics review or the need for informed consent. The QOR is managed by Cancer Alliance Queensland, with the main purposes of quality assurance and supporting clinician‐led research to improve patient outcomes. It brings together a wide range of data items from multiple sources, including demographic information, clinical characteristics, and treatment details. Data records for each person are consolidated through a series of complex linkage processes.

Follow‐up on treatment and mortality status was available for all members of the study cohort through to 31 December 2023. For the purposes of this study, only first‐line chemotherapy was considered.

The QOR does not routinely receive information on oral systemic therapy, which raises the prospect that some people treated with GEM may have received other chemotherapy drugs (such as Capecitabine) in tablet form, especially those from rural and remote areas. As the addition of oral chemotherapy has been shown to increase survival compared to GEM monotherapy, [18] a manual chart review was performed for people who received GEM and survived for longer than six months. Anyone who received additional oral chemotherapy was removed from the study cohort.

Patients who received other chemotherapy regimens or for whom the regimen was unknown/not stated were omitted. Note that not all facilities report the specific regimen administered to QOR, with this being more prevalent among private facilities. For this reason, the study cohort was limited to people who received chemotherapy at a public hospital or at an Icon Cancer Centre, where the required information was more widely available. People treated at all other private facilities were excluded due to insufficient details about the chemotherapy received.

The First Nations status for each person was determined by whether they self‐identified as being an Aboriginal and/or Torres Strait Islander. An algorithm‐based approach, building on work done in Western Australia [19], was used to enhance the accuracy of First Nations status using the multiple data sources feeding into the QOR. Stage at diagnosis, based on the eighth edition of the TNM classification system [20], was obtained by manual examination of each person's medical record if it was not already specified. Other key variables included remoteness of residence (categorised as major city, inner regional, outer regional and remote/very remote according to the Australian Statistical Geography Standard (ASGS), Edition 3 [21]), area‐based socio‐economic status (grouped as disadvantaged (deciles 1 and 2), middle SES (deciles 3–8) and advantaged (deciles 9 and 10) using the Index of Relative Socio‐Economic Advantage and Disadvantage [22]), and the number of comorbidities [23] (including conditions reported in any hospital admission within Queensland between 1 year before or after the cancer diagnosis).

### 2.2 Analyses

Chi‐square tests were used to evaluate possible differences in the distribution for each variable of interest by the chemotherapy regimen received. Demographic and clinical characteristics associated with receiving a particular type of chemotherapy were further examined using multivariable Poisson regression modelling. Results were expressed in terms of the adjusted relative likelihood (RL) compared to the reference category for each data item (the most common category was used as the reference unless otherwise stated). Median time and interquartile range (IQR) from diagnosis to the commencement of chemotherapy was also calculated by regimen, place of residence, and area‐based socio‐economic status, with the equality of the medians assessed using a nonparametric K‐sample test.

Observed survival by each variable of interest was estimated using the Kaplan–Meier method, irrespective of the cause of death. Survival time was calculated from the date of diagnosis until the date of death, the censoring date (31 December 2023) or three years after diagnosis, whichever occurred first. Differences in survival were formally assessed by fitting a flexible parametric survival model adjusted for personal and clinical covariates, including the type of chemotherapy regimen, and expressed as the excess mortality hazard ratio (HR) at three years after diagnosis. Flexible parametric modelling allows for nonproportional hazards through the use of cubic splines, thus avoiding the assumptions about the shape of the baseline hazard function [24].

Point estimates are presented with the associated 95% confidence intervals (95% CIs) and *p*‐values where relevant. Statistical significance was set at *p* ≤ 0.05. All analyses were conducted using Stata/MP for Windows version 18.0 (StataCorp LLC, College Station, Texas).

## 3 Results

### 3.1 Study Cohort

A total of 3472 residents were diagnosed with pancreatic cancer in Queensland between 2018 and 2022. People who had surgery were excluded (*n* = 540, 16%), as were those who did not have chemotherapy (*n* = 1643, 47%), patients who were treated at a private hospital other than an Icon facility (*n* = 427, 12%), those who did not have one of the three standard chemotherapy regimens of interest or where the type of first‐line chemotherapy was not recorded (*n* = 60, 2%), or pancreatic tumours that were not classified as adenocarcinomas (*n* = 36, 1%). The study cohort was comprised of the remaining 766 people (22% of all pancreatic cases) who had an unresected pancreatic adenocarcinoma and were treated with first‐line mFFX, GnP, or GEM—see Figure S1. Patients treated at Icon Cancer Centres comprised 30% (*n* = 165 of 551) of people with an unresected pancreatic adenocarcinoma who received chemotherapy at a private facility, and of these, 94% (*n* = 155) had information on the type of chemotherapy.

Just over half (*n* = 429, 56%) of the study cohort were male, and the overall median age at diagnosis was 67 years (IQR 60–74 years). Of the population groups of interest, 4% (*n* = 31) identified as a First Nations person, 11% (*n* = 88) lived in outer regional, remote or very remote parts of Queensland, and 30% (*n* = 232) were from disadvantaged socio‐economic areas. The majority of the study cohort presented with metastatic disease (*n* = 526, 69%), and approximately one‐third (*n* = 241, 31%) had two or more comorbidities. For the reasons outlined in the Methods section above, most were treated in a public hospital (*n* = 611, 80%).

### 3.2 Characteristics Associated With Type of Chemotherapy

GnP was the most common chemotherapy regimen (*n* = 450, 59%), followed by mFFX (*n* = 204, 27%) and GEM (*n* = 112, 15%). Median age by chemotherapy regimen varied widely from 61.5 years for mFFX to 68 years for GnP and 72.5 years for GEM (*p* < 0.001). Significant univariate differences in receipt of the various regimens were identified for age group at diagnosis (*p* < 0.001), First Nations status (*p* = 0.03), remoteness of residence (*p* = 0.001), area‐based socio‐economic status (*p* = 0.03), number of comorbidities (*p* = 0.01), stage at diagnosis (*p* < 0.001) and facility type (*p* < 0.001)—see Table 1.

**TABLE 1:** Selected chemotherapy regimens^a^ ^,^ ^b^ for unresected pancreatic adenocarcinoma by key patient and clinical characteristics, Queensland, 2018–2022.

Characteristic of interest	Total	mFFX		GnP		GEM	
	*n*	*n*	Row %	*n*	Row %	*n*	Row %
All patients^c^	766	204	26.6	450	58.8	112	14.6
Sex (*p* = 0.09)							
Males	429	127	29.6	245	57.1	57	13.3
Females	337	77	22.9	205	60.8	55	16.3
Age group at diagnosis (*p <* 0.001)							
< 65 years old	308	118	38.3	156	50.7	34	11.0
65–74 years old	288	70	24.3	192	66.7	26	9.0
75+ years old	170	16	9.4	102	60.0	52	30.6
First Nations status (*p =* 0.03)							
Aboriginal and/or Torres Strait Islander	31	13	41.9	11	35.5	7	22.6
Other Queenslanders	735	191	26.0	439	59.7	105	14.3
Remoteness of residence (*p* = 0.001)							
Major city	514	134	26.1	322	63.6	58	11.3
Inner regional	164	47	28.7	86	52.4	31	18.9
Outer regional/remote	88	23	26.1	42	47.7	23	26.1
Area‐based socio‐economic status (*p* = 0.03)							
Advantaged	91	34	37.4	50	54.9	7	7.7
Middle	443	109	24.6	271	61.2	63	14.2
Disadvantaged	232	61	26.3	129	55.6	42	18.1
Number of comorbidities (*p* = 0.01)							
None	332	102	30.7	194	58.4	36	10.8
One	193	54	28.0	109	56.5	30	15.5
Two or more	241	48	19.9	147	61.0	46	19.1
Stage at diagnosis (*p* < 0.001)							
I	44	16	36.4	21	47.7	7	15.9
II	47	20	42.6	19	40.4	8	17.0
III	125	46	36.8	56	44.8	23	18.4
IV	526	110	20.9	347	66.0	69	13.1
Not specified	24	12	50.0	7	29.2	5	20.8
Facility type (*p* < 0.001)							
Public	611	138	22.6	374	61.2	99	16.2
Private	155	66	42.6	76	49.0	13	8.4

Abbreviations: GEM, gemcitabine only; GnP, gemcitabine with nab‐paclitaxel; mFFX, oxaliplatin, irinotecan and fluorouracil.

^a^ First line chemotherapy only.

^b^ Excluding oral chemotherapy.

^c^ Includes people with pancreatic cancer who received one of the main three chemotherapy regimens (FFX, GnP or GEM) at any public hospital or selected private hospitals in Queensland.

**FIGURE 1:**
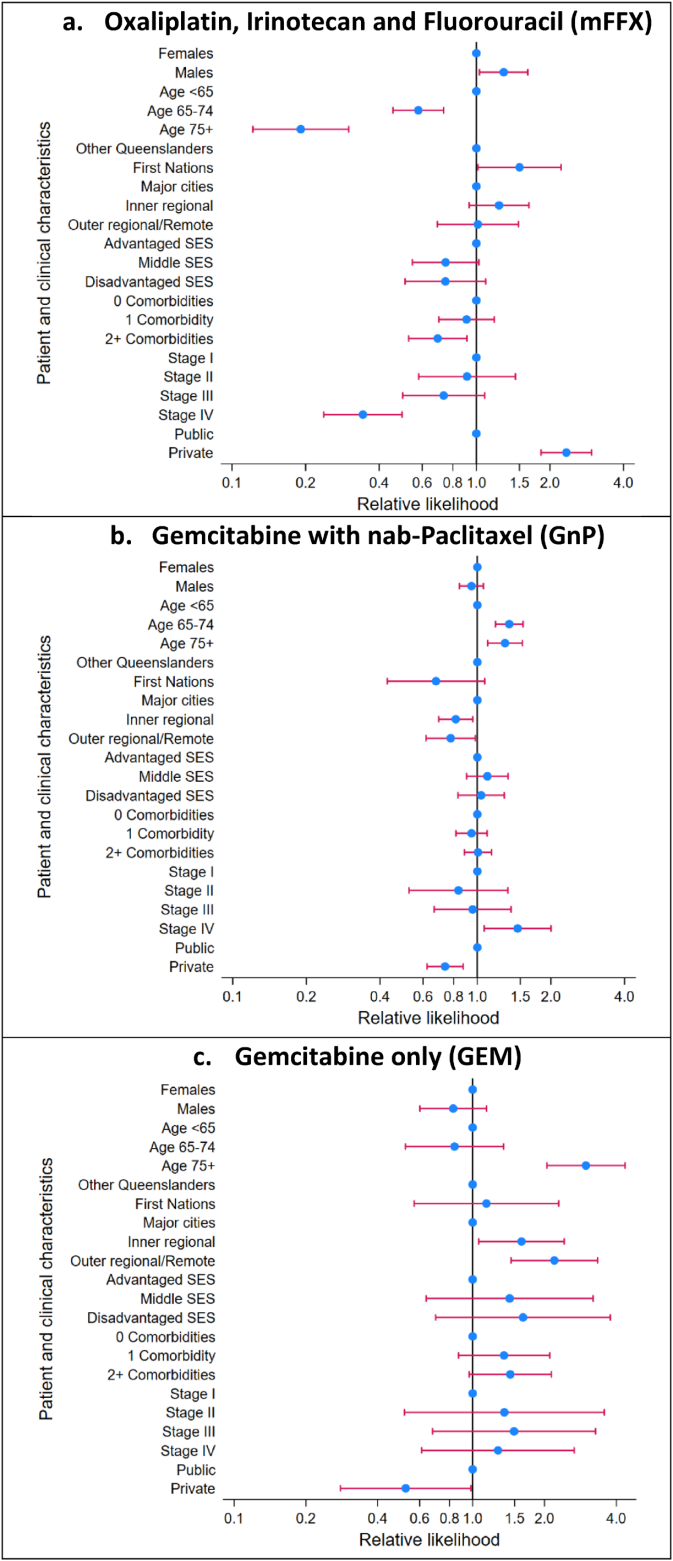
Relative likelihood of receiving chemotherapy regimens for unresected pancreatic adenocarcinoma by patient, clinical and facility characteristics, Queensland, 2018–2022.

Multivariable analysis revealed that people were far less likely to receive mFFX if they were aged 75 years and over (RL = 0.19 compared to the under 65 age group, 95% CI 0.12–0.30; *p* < 0.001), had stage IV disease (RL = 0.34 compared to stage I, 95% CI 0.24–0.50; *p* < 0.001) or had 2 or more comorbidities (RL = 0.69 compared to no comorbidities, 95% CI 0.53–0.91; *p* = 0.009), while people treated at an Icon Cancer Centre were more than twice as likely to receive mFFX than those treated in public hospitals (RL = 2.33, 95% CI 1.84–2.96; *p* < 0.001)—Figure 1. First Nations people were also more likely to be treated with mFFX compared to other Queensland residents (RL = 1.50, 95% CI 1.01–2.22; *p* = 0.04), although the estimate was based on a small number of people and should be interpreted with due caution. The adjusted effects of neither remoteness of residence (*p* = 0.31) or area‐based socio‐economic status (*p* = 0.18) were statistically significant.

In contrast to mFFX, being treated with the GnP regimen was more likely for those aged 65–74 (RL = 1.35 compared to the under 65 age group, 95% CI 1.19–1.54; *p* < 0.001) or with stage IV disease (RL = 1.46 compared to stage I, 95% CI 1.07–2.00; *p* = 0.02), but was less commonly used for people from outer regional/remote areas compared to major cities (RL = 0.78, 95% CI 0.62–0.98; *p* = 0.03) or in the selected private facilities (RL = 0.74, 95% CI 0.62–0.87; *p* < 0.001). Usage of the GEM regimen was by far highest for the 75 and over age group (RL = 2.98 compared to the under 65 age group, 95% CI 2.05–4.35; *p* < 0.001) or those from outer regional/remote areas (RL = 2.20 compared to people living in major cities, 95% CI 1.45–3.34; *p* < 0.001) and was again lower in private compared to public facilities (RL = 0.52, 95% CI 0.28–0.98; *p* = 0.04).

### 3.3 Time From Diagnosis to First Chemotherapy

**FIGURE 2:**
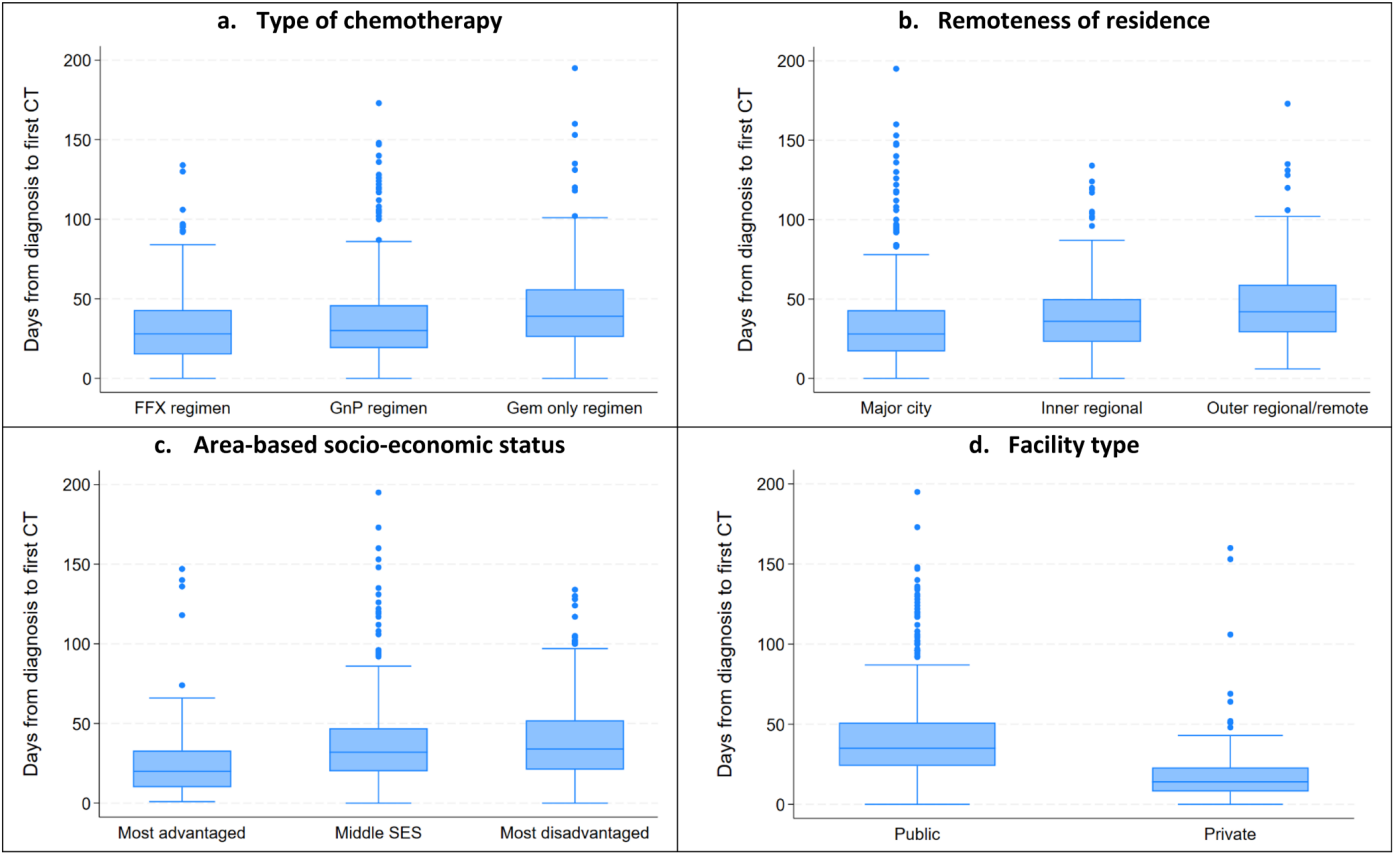
Days from diagnosis to start of chemotherapy for unresected pancreatic adenocarcinoma by (a) type of chemotherapy, (b) remoteness of residence, (c) area‐based socio‐economic status and (d) facility type, Queensland, 2018–2022. Excludes oral chemotherapy. *Y*‐axis truncated at 200 days. Private facilities include Icon Cancer Centres only. GEM, gemcitabine only; GnP, paclitaxel with gemcitabine; MFFX, oxaliplatin, irinotecan and fluorouracil.

The median time from diagnosis to date of first chemotherapy was 32 days (IQR 19–49 days) across the entire study cohort. There was significant variation by the type of chemotherapy, from a median of 28 days for mFFX to 40 days for GEM (*p* = 0.003)—see Figure 2. Differences in the median time to chemotherapy were also found by remoteness of residence, ranging from 28 days for people from major cities to 42 days in outer regional/remote localities, and by area‐based socio‐economic status, ranging from 20 days in the most advantaged areas to 34 days in the most disadvantaged areas (both *p* < 0.001). Some of these differences remained even after stratification by type of chemotherapy; for example, time to treatment with GnP varied from 27.5 days in major cities to 42 days in outer/regional/remote regions (*p* < 0.001) and from 14.5 days in the most advantaged areas to 34 days in the most disadvantaged areas for those who received first‐line mFFX (*p* = 0.001). A strong overall disparity in time from diagnosis to date of first chemotherapy was also found by facility type (median of 14 days for Icon private facilities compared to 36 days for public hospitals; *p* < 0.001), with a similarly large difference persisting irrespective of the chemotherapy regimen used (in particular, 13.5 days vs. 37 days, respectively, for mFFX; *p* < 0.001).

### 3.4 Radiotherapy

Almost one‐quarter (*n* = 178, 23%) of the total study cohort had radiotherapy in addition to chemotherapy. Radiotherapy was far more common in conjunction with mFFX (*n* = 74 of 204, 36%) than either GnP (*n* = 84 of 450, 19%) or GEM (*n* = 20 of 112, 18%). In most cases where radiotherapy was received, it was given subsequent to chemotherapy (*n* = 162 of 178, 91%).

### 3.5 Survival

Only 7% (*n* = 57) of the study cohort remained alive as of 31 December 2023. Median all‐cause survival time was 10 months (IQR 5–15 months) and ranged from 8 months for GEM to 9 months for GnP and slightly over 12 months for mFFX.

**FIGURE 3:**
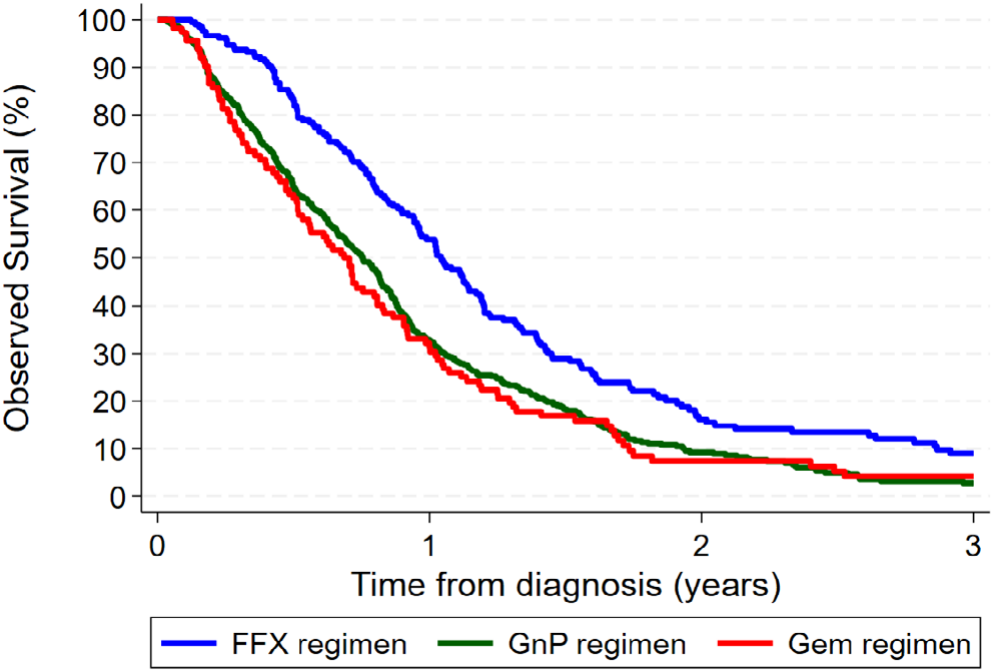
Three‐year Kaplan–Meier all‐cause survival curves for unresected pancreatic adenocarcinoma by type of chemotherapy, Queensland, 2018–2022. Includes people with pancreatic cancer who received one of the main three chemotherapy regimens (FFX, GnP or GEM). Excludes oral chemotherapy. Follow‐up for survival was available to 31 Dec 2023 for all patients. GEM, gemcitabine only; GnP, paclitaxel with gemcitabine; MFFX, oxaliplatin, irinotecan and fluorouracil.

Three‐year survival was estimated at 5% (95% CI 3%–7%) overall, varying from 3% (95% CI 1%–5%) for people treated with GnP and 4% (95% CI 1%–10%) for GEM to 9% (5%–14%) for mFFX (*p* < 0.001)—see Figure 3 and Figure S2. After multivariable analysis, a significant disparity in survival remained by type of chemotherapy (*p* = 0.005), with mortality within three years of diagnosis being 30% and 53% higher for the GnP and GEM regimens, respectively, compared to the mFFX regimen (Table 2). Survival was also lower for First Nations people compared to other Queensland residents (HR = 1.60, 95% CI 1.10–2.33; *p* = 0.01), those with 2 or more comorbidities compared to no comorbidities (HR = 1.37, 95% CI 1.14–1.64; *p* = 0.001), stage IV disease compared to stage I (HR = 2.28, 95% CI 1.60–3.25; *p* < 0.001), and to a lesser extent for people treated in public hospitals rather than at Icon private facilities (HR = 1.24, 95% CI 1.00–1.54; *p* = 0.05). No significant differences in survival were observed by sex (*p* = 0.12), age group at diagnosis (*p* = 0.32), remoteness of residence (*p* = 0.43) or area‐based socioeconomic status (*p* = 0.50).

**TABLE 2:** Results of multivariable flexible parametric survival model for unresected pancreatic adenocarcinoma, Queensland, 2018–2022.^a^

Covariate	*n*	Adjusted excess mortality HR (95% CI)	*p*
Chemotherapy regimen^c^ (*p* = 0.005)			
mFFX	204	1.00	
GnP	450	1.30 (1.07–1.59)	0.009
GEM	112	1.53 (1.17–2.01)	0.002
Sex (*p* = 0.12)			
Males	429	1.13 (0.97–1.32)	0.12
Females	337	1.00	
Age group at diagnosis (*p* = 0.32)			
< 65 years old	308	1.00	
65–74 years old	288	0.96 (0.81–1.15)	0.66
75+ years old	170	1.13 (0.91–1.41)	0.27
First Nations status (*p* = 0.01)			
Aboriginal and/or Torres Strait Islander	31	1.60 (1.10–2.33)	0.01
Other Australian	735	1.00	
Remoteness of residence (*p* = 0.43)			
Major city	514	1.00	
Inner regional	164	1.12 (0.92–1.38)	0.23
Outer regional/remote/very remote	88	1.11 (0.86–1.42)	0.43
Area‐based socioeconomic status (*p* = 0.50)			
Advantaged	91	1.00	
Middle	443	0.92 (0.71–1.19)	0.51
Disadvantaged	232	0.85 (0.64–1.13)	0.26
Number of comorbidities^b^ (*p* = 0.003)			
None	332	1.00	
One	193	1.12 (0.92–1.35)	0.25
Two or more	241	1.37 (1.14–1.64)	0.001
Stage (*p* < 0.001)			
I	44	1.00	
II	47	1.11 (0.70–1.74)	0.66
III	125	1.17 (0.80–1.72)	0.42
IV	526	2.28 (1.60–3.25)	< 0.001
Unknown	24	1.06 (0.58–1.94)	0.84
Facility type (*p* = 0.05)			
Public	155	1.24 (1.00–1.54)	0.05
Private^d^	611	1.00	

Abbreviations: CI, confidence interval; GEM, gemcitabine only; GnP, gemcitabine with nab‐paclitaxel; HR, hazard ratio; mFFX, oxaliplatin, irinotecan and fluorouracil.

^a^ All cause survival. Follow‐up for survival was available to 31 Dec 2023 for all patients.

^b^ Comorbidities were based on the Charlson Comorbidity Index (excluding second primary cancers) and include clinical conditions that have the potential to significantly affect the prognosis of a patient with cancer, coded in any admission episode between 12 months before or after the date of cancer diagnosis.

^c^ Excluding any oral chemotherapy.

^d^ Includes Icon Cancer Centres only.

## 4 Discussion

This study highlights the challenges of providing equitable care for Queensland residents diagnosed with pancreatic cancer, in a relatively small population (estimated to be 5.5 million at 31 December 2023) [25] spread across a very large geographical area (1.7 million square kilometres). The decision regarding the choice of first‐line chemotherapy for unresectable and/or metastatic disease is informed by factors such as treatment intent and the anticipated ability of the patient to tolerate expected side effects [26] Regimens that involve a combination of drugs (e.g., mFFX and GnP) are intended to improve survival but are associated with an increased risk of adverse events [27] which impacts the person's quality of life. A meta‐analysis conducted by Chen et al. [28] demonstrated comparable toxicity profiles for mFFX and GnP, but they were associated with significantly increased risks of neutropenia, nausea, diarrhoea and anaemia compared to GEM.

Other important factors which need to be taken into account when deciding the most suitable regimen include geographic and financial considerations. Most tertiary public hospitals in Queensland that offer comprehensive cancer services are concentrated in the south‐east corner of the state. Nearby access to chemotherapy for people living in rural and remote communities is typically limited to intermittent day services only, requiring a choice between travelling hundreds of kilometres to receive more complex regimens or else opting for simpler treatments available closer to home. For example, the limited pharmacological stability of prepared GnP compared to GEM [29] combined with a lack of pharmaceutical compounding facilities to prepare and provide complex protocols, is likely to influence the decision to treat people living in rural or remote areas with GEM rather than GnP, as reflected in our results.

From a financial perspective, people with pancreatic cancer are only funded by the Federal government for first‐line GnP in Australia under the Pharmaceutical Benefits Scheme. This may explain why GnP was by far the most common choice of first‐line chemotherapy within our study cohort (59% vs. 27% for mFFX). A study on unresectable pancreatic cancer from British Columbia, Canada, diagnosed between 2014 and 2016 [30] reported a much different distribution by type of chemotherapy compared to Queensland, with 41% of people treated with mFFX, 39% with GnP, and 20% with GEM. Younger people and those with better performance status were more likely to receive mFFX according to the Canadian study [30] in line with our findings.

We also observed that patients in the selected private facilities were significantly more likely to be treated with mFFX than those attending public hospitals, even after adjustment for patient and clinical characteristics. Further, people treated at public hospitals waited almost three times as long to receive chemotherapy compared to those at private Icon Cancer Centres. Some of this delay could be explained by longer wait times for insertion of central lines, required for mFFX but also needed if patients have poor venous access. This points to apparent inequities in the type and timeliness of treatment received based on the ability to afford private health cover, which in turn may affect a person's chances of survival.

Despite the outcome for unresectable pancreatic cancer remaining dismal overall, the type of chemotherapy nonetheless had a significant bearing on survival; the risk of mortality within three years of diagnosis was 30% higher for GnP, and more than 50% higher for GEM, compared to mFFX in Queensland. Wang et al. [30] reported median survival of 11 months for GnP and 14 months for mFFX among people treated at six cancer centres in British Columbia during 2014–2015. Similarly, a multi‐institutional international review [31] involving studies that were published prior to 2020 revealed median overall survival of 11 and 15 months for GnP and mFFX for unresectable locally advanced pancreatic cancer, respectively. These findings were somewhat longer than our results for those two regimens (9 months for GnP and 12 months for mFFX), possibly reflecting differences in study methodology, such as patient selection being hospital‐based versus population‐based. In contrast, median survival following first‐line treatment with GEM in Canada (4 months) [30] was only half as long or less compared to Queensland (8 months) and the international analysis (10 months) [31].

Aboriginal and/or Torres Strait Islander people in the study cohort tended to be more likely to receive mFFX (with the caveat that the result is based on a small number of patients). However, their survival was significantly worse, with a 60% increased risk of mortality compared to other Queensland residents after adjustment for other potential diagnostic factors. The reasons for this specific result are not clear, although it does reflect the consistently poorer outcomes for Aboriginal and/or Torres Strait Islander people in Australia with several other types of cancer [32, 33, 34]. A recent audit into health outcomes in general for First Nations people in Queensland concluded that social, environmental and underlying health issues were potential factors that contributed to the disparity, as well as a lack of access to and overall trust in the Western healthcare system, which may lead to poor attendance and suboptimal adherence with treatment regimens [35].

A strength of this study was the availability of high‐quality linked data available from the QOR. Stage at diagnosis was assigned for 97% of the study cohort; many of these cases were able to be manually staged using data from medical imaging and medical records, including multidisciplinary team meetings. Some potential limitations must also be taken into account when interpreting our results. Only 30% of patients who were treated at private facilities in Queensland were considered for inclusion in the study due to the type of chemotherapy not being consistently recorded (apart from Icon Cancer Centres); it is therefore not clear to what extent our results are generalisable to all private facilities. There is also a possibility that some patients may have commenced chemotherapy with a neoadjuvant intent, particularly those diagnosed with earlier‐stage disease who were ultimately deemed ineligible for surgery due to disease progression or poor performance status. We are not able to identify which people this may have affected nor quantify the impact on our results.

With notable differences in survival dependent on which first‐line systemic therapy is received, it is imperative that equitable access to treatment is sought for patients diagnosed with unresectable pancreatic ductal adenocarcinoma. Whilst challenges exist, as discussed, including drug stability and locality to a chemotherapy treatment unit, improving timely access and mechanisms to overcome other barriers need to be explored. The rapid uptake of telehealth allows safer monitoring of patients undergoing cancer care in rural or remote areas, [36] and should become ingrained in any service treating these patients. Expanding a digital health ecosystem, as well as the development of a national framework of networked comprehensive cancer care centres designed to extend expertise to patients and their families living in regional, rural and remote areas, have been identified as key pillars in the Australia Cancer Plan [37] to reduce inequitable cancer care. It is hoped that these initiatives may help to reduce any disparities in the treatments offered between metropolitan or affluent patients and those from regional and remote localities or areas with lower socio‐economic status.

## Author Contributions

**Danny R. Youlden:** formal analysis, writing – original draft. **Bryan A. Chan:** supervision, writing – review and editing. **Jon Clark:** data curation, writing – review and editing. **Victoria K. Donoghue:** validation, writing – review and editing. **Michael J. Allen:** conceptualization, supervision, writing – review and editing.

## Conflicts of Interest

The authors declare no conflicts of interest.

## Supporting information


**Figure S1:** Flow diagram for selection of the study cohort. Percentages shown are of all Queensland residents diagnosed with pancreatic cancer between 2018 and 2022.

**Figure S2:** Three‐year Kaplan–Meier survival curve for unresected pancreatic adenocarcinoma by selected patient and clinical characteristics, Queensland, 2018–2022. All cause survival. Follow‐up for survival was available to 31 Dec 2023 for all patients. Private facilities include Icon Cancer Centres only.

## Data Availability

The unit record data used in this study are not publicly available to protect patient privacy and confidentiality. Deidentified data may be available from the corresponding author on reasonable request.
