# Supplementary figures and images for: Treatment and Survival for Unresectable Pancreatic Adenocarcinoma in Queensland, Australia, 2018–2022
Source: Cancer Med. 2025 Sep 8;14(17):e71226. doi: 10.1002/cam4.71226 (PMC12415588; doi:10.1002/cam4.71226)

**Supplementary Figure 1**

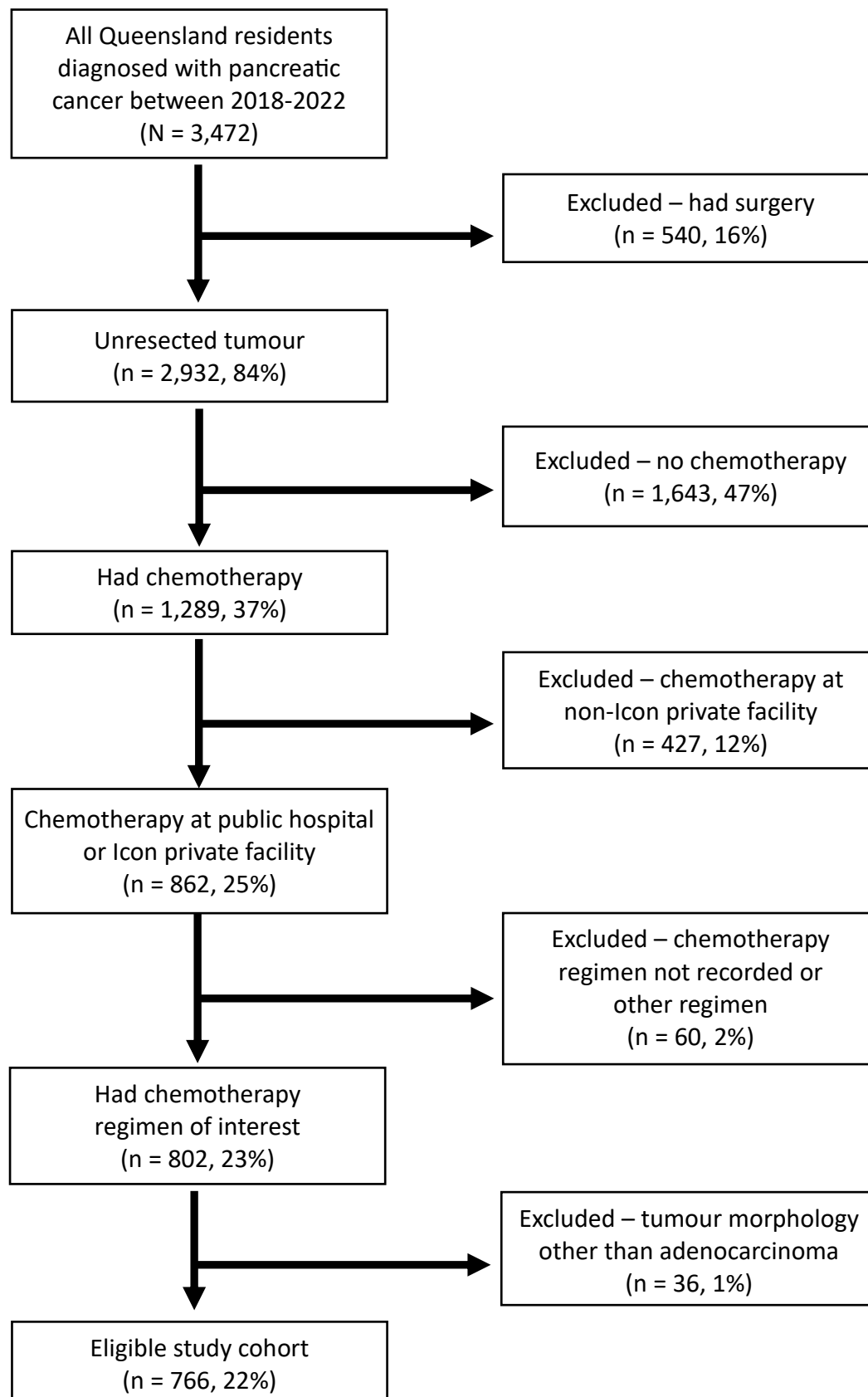

Supplement: Supplementary file 1 — Figure S1: Flow diagram for selection of the study cohort. Percentages shown are of all Queensland residents diagnosed with pancreatic cancer between 2018 and 2022. [file CAM4-14-e71226-s002.pdf]

**Supplementary Figure 2**

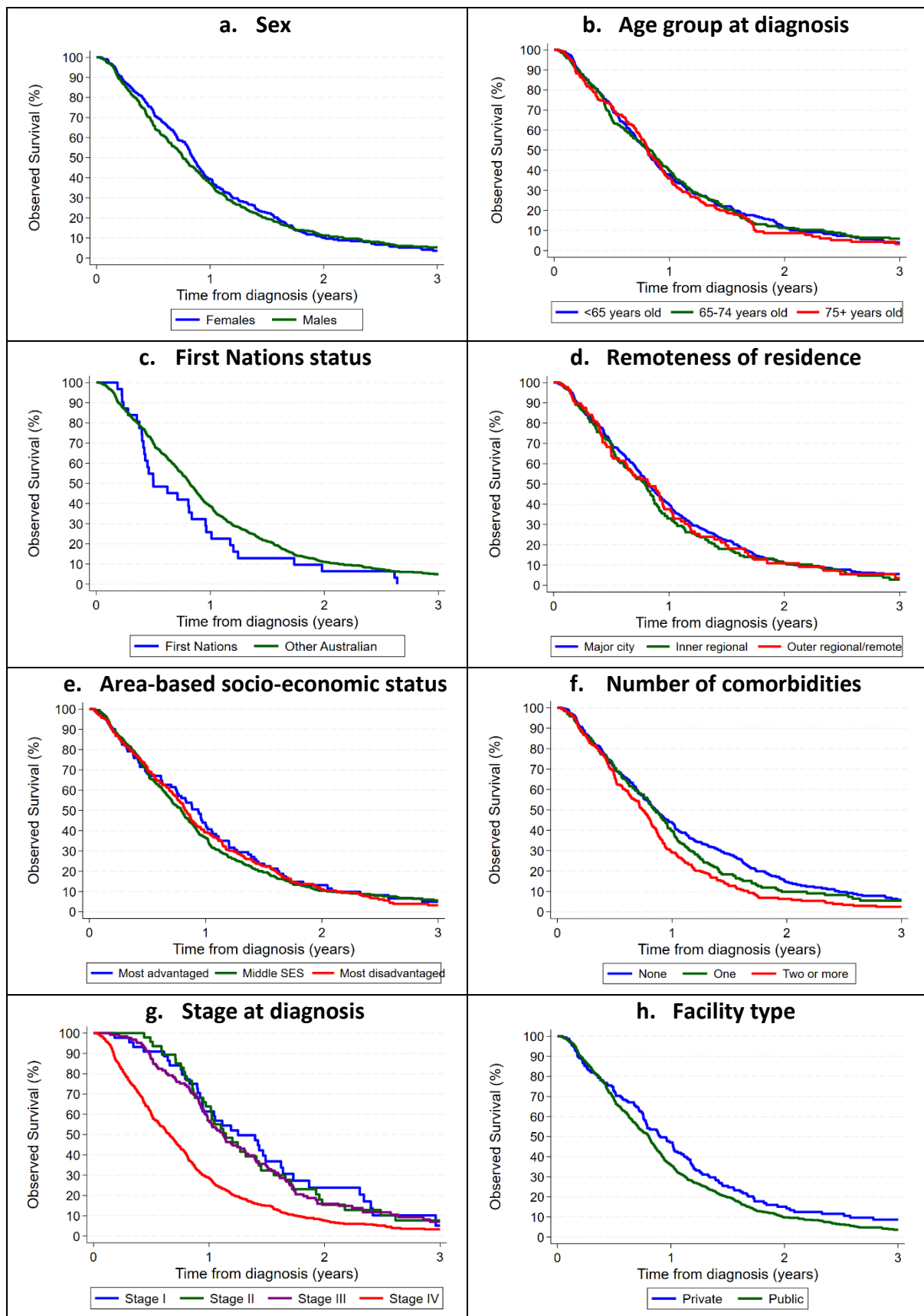

Supplement: Supplementary file 2 — Figure S2: Three‐year Kaplan–Meier survival curve for unresected pancreatic adenocarcinoma by selected patient and clinical characteristics, Queensland, 2018–2022. All cause survival. Follow‐up for survival was available to 31 Dec 2023 for all patients. Private facilities include Icon Cancer Centres only. [file CAM4-14-e71226-s001.pdf]
